# UK psychiatrists’ experience of withdrawal of antipsychotics prescribed for challenging behaviours in adults with intellectual disabilities and/or autism

**DOI:** 10.1192/bjo.2020.97

**Published:** 2020-09-17

**Authors:** Shoumitro Deb, Tom Nancarrow, Bharati Limbu, Rory Sheehan, Mike Wilcock, David Branford, Ken Courtenay, Bhathika Perera, Rohit Shankar

**Affiliations:** Faculty of Medicine, Department of Brain Sciences, Imperial College London, UK; University of Exeter Medical School, UK; Faculty of Medicine, Department of Brain Sciences, Imperial College London, UK; Division of Psychiatry, University College London, UK; Royal Cornwall Hospital, Truro, UK; NHS England, Leicester, UK; Faculty of Intellectual Disabilities, Royal College of Psychiatrists, London, UK; Haringey Learning Disability Partnership, Barnet, Enfield and Haringey Mental Health NHS Trust, London, UK; University of Exeter Medical School, UK

**Keywords:** Intellectual disabilities, antipsychotics, withdrawal, adults, STOMP

## Abstract

**Background:**

A high proportion of adults with intellectual disabilities are prescribed off-licence antipsychotics in the absence of a psychiatric illness. The National Health Service in England launched an initiative in 2016, ‘Stopping over-medication of people with a learning disability [intellectual disability], autism or both’ (STOMP), to address this major public health concern.

**Aims:**

To gain understanding from UK psychiatrists working with adults with intellectual disabilities on the successes and challenges of withdrawing antipsychotics for challenging behaviours.

**Method:**

An online questionnaire was sent to all UK psychiatrists working in the field of intellectual disability (estimated 225).

**Results:**

Half of the 88 respondents stated that they started withdrawing antipsychotics over 5 years ago and 52.3% stated that they are less likely to initiate an antipsychotic since the launch of STOMP. However, since then, 46.6% are prescribing other classes of psychotropic medication instead of antipsychotics for challenging behaviours, most frequently the antidepressants. Complete antipsychotic discontinuation in over 50% of patients treated with antipsychotics was achieved by only 4.5% of respondents (*n* = 4); 11.4% reported deterioration in challenging behaviours in over 50% of patients on withdrawal and the same proportion (11.4%) reported no deterioration. Only 32% of respondents made the diagnosis of psychiatric illness in all their patients themselves. Family and paid carers’ concern, lack of multi-agency and multidisciplinary input and unavailability of non-medical psychosocial intervention are key reported factors hampering the withdrawal attempt.

**Conclusions:**

There is an urgent need to develop national guidelines to provide a framework for systematic psychotropic drug reviews and withdrawal where possible.

Individuals with intellectual disabilities are at a higher risk of developing challenging behaviours (also known as behaviours that challenge). Although challenging behaviours have been reported in up to 62%^1–4^ of adults with intellectual disabilities, more severe challenging behaviour is reported in a smaller proportion of people (around 18%).^5^ Aggression (11%) towards other individuals and objects and self-injurious behaviour are the most common forms of challenging behaviour.^6^ Challenging behaviours in general, and aggression in particular, pose a major management problem and are a significant barrier to social integration, and may lead to caregiver stress, community placement breakdown and use of restrictive practices. A thorough person-centred assessment with multidisciplinary input is vital for successful management. The assessment should take a biopsychosocial approach by incorporating assessments of the behaviour, the person showing the behaviour, and medical, psychiatric/psychological and social/environmental factors.^4^

Both medications^7^ and non-medication-based psychosocial interventions^8^ are used to manage challenging behaviours. However, national^9^ and international^10^ guidelines recommend the use of psychosocial intervention first and the use of medication only when the psychosocial interventions have failed and the persons themselves or people around them are at a serious risk of harm. Nevertheless, psychotropic medications are prescribed widely (32–85%) to people with intellectual disabilities, the average being around 50–63%.^11^ Most widely used among psychotropics are the antipsychotics, which are received by 21% of adults with intellectual disabilities.^11^ This compares with the rate of antipsychotic use of <1% in the general population.^12^ Antipsychotics are often used off-licence in intellectual disability in the absence of a mental illness (36–71%), primarily to manage challenging behaviours.^13^ This shows that the national and international guidelines need a structure and a support framework for implementation.

The off-licence use of antipsychotics in people with intellectual disabilities is a major cause of public health concern, as it has been suggested that every year in England 35 000 adults with intellectual disabilities receive psychotropic medication unnecessarily,^14^ and the long-term use of antipsychotics carries the risk of medication-related adverse events that could affect the person's quality of life.^15^ As a result, in 2016 the National Health Service (NHS) in England has embarked on a major campaign, ‘Stopping over-medication of people with a learning disability [intellectual disability], autism or both’ (STOMP).^16^

National^9^ and international^10^ guidelines recommend that psychotropic medication should be reviewed regularly, and consideration given to reduction/discontinuation or use for shortest time possible. One practical way to reduce overmedication in this population is to withdraw psychotropics, particularly antipsychotics, by tapering dosage. Studies from the UK and The Netherlands show that it is possible to totally discontinue antipsychotics in 25–46.5% of patients after long-term use and to achieve a dose reduction of over 50% in a further 11–19% of individuals.^17–21^

A number of factors have been shown to affect withdrawal. For example, one of us (D.B)^17^ found that a lower dose of antipsychotics, minimal psychopathology, and the lack of aggression, stereotypy and hyperactivity at baseline helped with the withdrawal. de Kuijper and colleagues^19^ found that female gender, a lower rate of baseline challenging behaviours and lower baseline antipsychotic dosage are in favour, and the presence of severe challenging behaviours, and autonomic and extrapyramidal symptoms at baseline are factors against a successful withdrawal. They also found that comorbid autism, a higher dose of antipsychotic, greater severity of challenging behaviours and higher akathisia scores, and more frequent worsening of health during withdrawal were associated with a lower incidence of complete discontinuation. These factors also affect reinstatement rates.

Although the withdrawal of antipsychotics shows improvement in challenging behaviours and quality of life among most people, in some it carries the risk of precipitating withdrawal symptoms that may manifest as challenging behaviours.^7^ However, other possibilities also need to be considered. For example, behaviour may deteriorate for reasons that are not at all related to the withdrawal process, so one should not automatically relate deterioration in behaviour to the dose reduction or withdrawal. Instead, a thorough assessment of behaviour should be carried out.^4^ Sometimes an underlying psychiatric disorder may be unmasked on withdrawal of psychotropic medication or dose reduction, particularly if the medication has been used for a long time. A careful assessment of mental state will be necessary, as making a diagnosis of a psychiatric disorder in a person with intellectual disabilities can be difficult.^4,22^ In some cases, previous behaviour may return on dose reduction or withdrawal. In these circumstances, a full functional assessment of the behaviour will be necessary, including the assessment of predisposing (e.g. genetic syndromes), precipitating (e.g. life events) and perpetuating (e.g. inappropriate environment) factors.^4^ Also, sometimes carers’ anxieties about medication withdrawal may be reflected in a heightened perception of severity of challenging behaviours on withdrawal.

The aim of the current study was to assess UK psychiatrists’ psychotropic prescribing practice for adults with intellectual disabilities who display challenging behaviours, with a focus on withdrawal of antipsychotics.

## Method

An online survey using the STROBE cross-sectional study model was developed through a consultation process by a core team of psychiatrists, pharmacists and academics working in the field of psychiatry of intellectual disability, with input from patient representative groups. The survey questions can be found in the supplementary material available at https://doi.org/10.1192/bjo.2020.97 (file Supplementary information 1).

The questionnaire with a cover letter was emailed to the UK Royal College of Psychiatrists’ intellectual disability regional representatives and training programme directors practising in the psychiatry of intellectual disability, who were asked to forward the questionnaire to the psychiatrists working within their respective regions. It was supported by the College's Faculty of Psychiatry of Intellectual Disability. The survey was open between 12 October 2019 and 29 February 2020 and three email reminders were used to encourage participation.

The survey took approximately 10 min to complete and contained the following main subsections:

- respondent characteristics, such as current medical position, place of practice, number of years practising psychiatry;
- the extent of attempts and the experience of reducing/withdrawing antipsychotic medication made by that clinician thus far, for example identifying the proportion of people with intellectual disabilities in whom respondents have completely withdrawn antipsychotics;
- prescribing habits since the introduction of the STOMP initiative, such as identifying whether respondents are now less likely to prescribe antipsychotics for challenging behaviours or are prescribing a different class of psychotropic instead of antipsychotics;
- the structures in place to support the withdrawal of antipsychotics prescribed for challenging behaviours, by looking at support available and resources developed or used during withdrawal process, use of outcome measures, confidence in withdrawing antipsychotics, etc.;
- the successes and challenges experienced in withdrawing antipsychotics prescribed for challenging behaviours, by looking at the barriers and challenges that hamper the withdrawal process, and extra resources required to improve antipsychotic prescribing practice for challenging behaviours, etc.

Most questions were assessed using multiple choice options or scales to aid the analysis. Free-text responses were used to gain data where multiple choice or sliding scales were not suitable and to gain qualitative data.

### Ethics and participation consent

No ethical approval was required as this was a survey of psychiatrists’ opinion and did not collect any individual patient data. All potential participants were advised that participation was voluntary, and their replies would be anonymised before analysis.

### Data analysis

Descriptive statistical analyses were carried out primarily to provide data on proportions, and chi-squared and Fisher's exact probability tests, as appropriate, were conducted for intergroup analysis. Qualitative data were analysed using thematic analysis and will be reported in a separate paper.

## Results

The dissemination of the survey relied on the regional representatives and training programme directors as third parties. We estimate that around 225 psychiatrists working in the specialty received the questionnaire, 88 (39%) of whom returned the completed questionnaire.

### Respondents’ characterises

Respondents’ characteristics are described in Table 1.

**Table 1:** Respondents’ characteristics (*n*=88)

*n*	%
Psychiatrists’ grade	
Consultant	87.5
Higher trainee	6.8
Specialty doctor	5.7
Region of practice
London	20.5
South West of England	15.9
South East of England	11.4
East Midlands	10.2
East of England	8
West Midlands	6.8
North East England	5.7
Scotland	5.7
Wales	4.5
Yorkshire and Humber	4.5
Northern Ireland	4.5
Other locations	2.2
Duration of practice in the psychiatry of intellectual disability
Less than 10 years	45.5
10–19 years	35.2
20–29 years	15.9

In total, 28.4% (*n* = 25) of respondents stated that they have a database of people with intellectual disabilities/autism spectrum disorder who have been prescribed antipsychotics in the absence of a severe mental illness.

### Initiation of antipsychotics for challenging behaviours

Just over half of the respondents (52.3%; *n* = 46) stated that they were less likely to initiate an antipsychotic for challenging behaviours since the launch of the STOMP initiative. Since the introduction of the STOMP initiative, 46.6% (*n* = 41) of participants are more often prescribing other classes of psychotropic medication instead of antipsychotics but 53.4% (*n* = 47) are not. Among the other classes of psychotropic prescribed by the 41 respondents, 23.8% reported prescribing antidepressants, 5.7% mood stabilisers, 5.7% benzodiazepines, 4.5% anti-epileptics and 3.8% anxiolytics.

### Withdrawing antipsychotics prescribed for challenging behaviours

Half of the participants started withdrawing antipsychotics more than 5 years ago, 18.2% started 3–5 years ago, 23.9% 1–3 years ago, 5.7% less than 1 year ago and 2.3% have not started withdrawing. Apart from antipsychotics, respondents also attempted to withdraw antidepressants (16%), benzodiazepines (13.3%), mood stabilisers (12.3%) and anti-epileptics (7.7%).

Over one-third of respondents (36.4%) attempted to withdraw antipsychotics in more than 50% of patients receiving the drugs for challenging behaviours, 15.9% in 26–50% and 28.4% in 1–25%. Only 1.1% of respondents did not attempt to withdraw antipsychotics in eligible patients. Withdrawal attempt data were not available for 17% of the respondents.

### Rates of withdrawal, dose reduction and reinstatement

Table 2 contains information on the proportion achieving a successful withdrawal and the rate of reinstatement. Only 4.5% (*n* = 4) of respondents achieved a complete withdrawal in over 50% of patients who were on antipsychotics inappropriately. The majority (60.2%) (*n* = 52) achieved this among 1–25%. A slightly better ratio was reported for over 50% dose reduction, achieved by 9.1% (*n* = 8) of respondents in over 50% of patients, compared with 15.9% (*n* = 14) in 26–50% of patients and 54.5% (*n* = 48) in 1–25% of patients. Reinstatement of antipsychotics was at its highest within the first 3–6 months but may have increased in some cases at 12-month follow-up.

**Table 2:** Proportions of patients with successful withdrawals and dose reductions and the rate of reinstatement (*n* = 88 respondents)

	Proportion of patients, %	Respondents	
		*n*	%
Achieved complete withdrawal from antipsychotics	0	9	10.2
	1–25	53	60.2
	26–50	9	10.2
	>50	4	4.5
	Not available	12	13.6
	Not applicable	1	1.1
Achieved >50% antipsychotic dose reduction	0	3	3.4
	1–25	48	54.5
	26–50	14	15.9
	>50	8	9.1
	Not available	15	17
Antipsychotic reinstated			
Within 3 months	0	20	22.7
	1–25	45	51.1
	26–50	14	15.9
	>50	8	9.1
	Not available	1	1.1
Within 6 months	0	22	25
	1–25	46	52.3
	26–50	11	12.5
	>50	8	9.1
	Not available	1	1.1
Within 12 months	0	31	35.2
	1–25	38	43.2
	26–50	7	8
	>50	11	12.5
	Not available	1	1.1

Twenty-six respondents (29.5%) who attempted antipsychotic withdrawal were unsuccessful in achieving a complete withdrawal in over 50% of patients, 25% (*n* = 22) were unsuccessful in 26–50% of patients and 26.1% (*n* = 23) were unsuccessful in 1–25%. However, 4.5% (*n* = 4) of respondents stated that they had never been unsuccessful in completely withdrawing antipsychotics; this information was not available for 14.8% (*n* = 13) of respondents.

### Changes in behaviour

A small proportion (11.4%) of respondents stated that behaviour had deteriorated in over 50% of patients in whom an antipsychotic withdrawal was attempted and the same proportion (11.4%) reported no deterioration in their patients on antipsychotic withdrawal. Additionally, 46.6% noted deterioration in behaviour in 1–25% of patients and 17% in 26–50%.

### Factors helping a successful withdrawal

Table 3 describes the reasons provided by the respondents that helped a successful withdrawal. These included low-dose antipsychotics (11.7%), antipsychotic monopharmacy (10.8%), first attempt at withdrawal (9.9%), antipsychotic polypharmacy (9.4%), polypharmacy of psychotropics (7.5%), experiencing side-effects of medication (7%), mild intellectual disability (6.6%) and living with family (6.1%).

**Table 3:** Factors affecting a successful withdrawal (*n* = 88 respondents)

Factor	Responses	
	*n*	%
Low-dose antipsychotics	25	11.7
Antipsychotic monopharmacy	23	10.8
First-time withdrawal attempt	21	9.9
Antipsychotic polypharmacy	20	9.4
No factors	19	8.9
Polypharmacy of psychotropics	16	7.5
Experiencing side-effects of medication	15	7
Mild intellectual disability	14	6.6
Living with family	13	6.1
Polypharmacy including physical health drugs and psychotropics	10	4.7
Psychotropic monopharmacy	9	4.2
High-dose antipsychotics	9	4.2
Other	5	2.3
Data not available	5	2.3
Moderate intellectual disability	2	0.9
Severe behaviours that concern	2	0.9
Severe intellectual disability	2	0.9
Male patient	1	0.5
Female patient	1	0.5
Mild behaviours that concern	1	0.5
Total	213	100

### Antipsychotics prescribed for psychiatric illness

In total, 12.5% of respondents reported that, in over 50% of their patients who were receiving antipsychotics, they were prescribed for an underlying psychiatric illness (compared with 13.6% for 26–50% of patients and 15.9% for 1–25% of patients). Therefore, none of these patients was considered for an antipsychotic withdrawal. However, 35% of respondents did not have any information available on this and 1.1% skipped this question.

From response to a subsequent question, it transpired that only 33.2% of respondents had made a diagnosis of mental illness in their patients themselves. A further 21.4% stated that the psychiatric diagnosis was made by a colleague, and 30.9% stated that the diagnosis was historical and gathered from patients’ case notes; 4.1% stated that the diagnosis was confirmed by a caregiver, and 7.3% stated that the diagnosis was gathered from the general practitioner's (GP's) case record. Among those respondents who did not consider antipsychotic withdrawal in the patients whom they thought had a psychiatric illness, 18.2% were extremely confident and 29.5% moderately confident in the diagnosis of the psychiatric illness.

### Outcome measures

Only one-third (33%, *n* = 29) of the respondents reported using outcome measures while withdrawing antipsychotics and 67% (*n* = 59) did not use any.

### Barriers and support requirement for a successful antipsychotic withdrawal

Table 4 presents the barriers and support requirements described by the respondents. Among the barriers, both paid carers’ and family carers’ resistance are rated high. Among the support requirement, multi-agency working and multidisciplinary team input were rated high.

**Table 4:** Barriers and support requirement for a successful antipsychotic withdrawal (*n* = 88 respondents)

Item	Respondents %
Barriers	
Resistance from support staff	22.4
Resistance from family carers	20.1
Lack of non-pharmacological psychosocial interventions	19.5
Lack of Multidisciplinary team support	12.8
Lack of national guidelines on a structure for withdrawal	7.6
Lack of pharmacist input	7
Reluctance of local GP to get involved	6.1
Lack of competency in withdrawing antipsychotics	4.7
Support required for a successful withdrawal programme	
Multiagency working	24.5
Multidisciplinary team input	22.3
Financial help for the patients and the local services	13.3
Nurse prescriber and other allied professionals input	11.2
Social worker input	9.4
Pharmacist input	8.2

GP, general practitioner.

### Intergroup analysis

Intergroup analysis showed no association between overall success in withdrawing antipsychotics and the professional level of the prescribing psychiatrist (consultant versus trainee) (*P* = 0.282), the number of years in practice (*P* = 0.254) and region of practice (*P* = 0.25). There was no association between the likelihood of initiating antipsychotics in response to STOMP and a psychiatrist's position (*P* = 0.864) or number of years in post (*P* = 0.093). A significantly higher proportion of respondents who received support from local services managed to achieve a complete antipsychotic withdrawal (*P* = 0.001; effect size 0.518).

## Discussion

Interestingly, almost one-third (28.4%) of the respondents reported that they have a database of patients with intellectual disabilities/autism spectrum disorder who have been prescribed antipsychotics in the absence of a severe mental illness. If this information reflects a true trend, and the quality of the databases is good, then it is encouraging, although eventually this figure needs to be 100% to facilitate the antipsychotic withdrawal process.

### Initiation of antipsychotics for challenging behaviours

About half of the responders stated that they are less likely to initiate antipsychotics for challenging behaviours since the launch of the STOMP pledge. Although this is a welcome move, a high proportion also stated that they are now prescribing other classes of psychotropic, in particular antidepressants and benzodiazipines, for this indication. This goes against the spirit of the STOMP pledge. However, the lack of non-pharmacological psychosocial support is likely to be one reason for this increase in prescription of non-antipsychotic psychotropics. Other studies have shown a similar trend in increase in antidepressant prescribing.^23,24^ However, it is also possible that some interpreted the question incorrectly and may have stated what other class of psychotropic other than antipsychotics they would prescribe if they had to use medication for challenging behaviours.

### Withdrawing antipsychotics prescribed for challenging behaviours

Half of the respondents started withdrawing antipsychotics more than 5 years ago. This figure is encouraging. It would have been interesting to understand whether those supporting withdrawal approach it in a systematic manner. This is difficult to infer owing to the lack of standards/guidelines. It is possible that more of those who are engaged in withdrawal have responded to the survey, thus creating a reporting bias. However, it also shows that many psychiatrists were involved in the withdrawal process even before the introduction of the STOMP initiative.

Just over one-third (36.4%) of respondents reported that they had tried withdrawal in over 50% of eligible patients (those who are prescribed antipsychotics for challenging behaviours) and about one-third (28.4%) tried this in 1–25% of patients. However, 1–25% is a wide range and it is difficult to know what proportion of respondents attempted withdrawal at the lower end of the range (say 1–5%).

### Total discontinuation versus dose reduction

Only a small number (*n* = 4) achieved a complete antipsychotic withdrawal in over 50% of patients (Table 2). Although total discontinuation was not possible in many cases, in some cases a partial withdrawal in the form of over 50% dose reduction was possible. This may be because behaviour deteriorated in some cases on dose reduction and therefore complete discontinuation was not possible. It is also possible that some of these will eventually achieve a complete discontinuation of antipsychotics as the dose reduction is continued in future. The other possibility is that, after complete discontinuation, in some cases antipsychotics were reinstated but, owing to stabilisation of behaviour, the ultimate dose remained lower than 50% of the original dose.

A greater proportion achieved complete discontinuation among only 1–25% of patients and we do not know what proportion of them achieved this at the lower end of the range (say 1–5%). There was no statistically significant association in the rate of successful withdrawal and the respondents’ region of practice, duration of practice and medical position. The reinstatement rate was highest within the first 3–6 months, which is expected.^17,19,20^ Previous withdrawal studies showed a reinstatement rate between 16 and 42%.^17,19,20^

### Withdrawing psychotropics other than antipsychotics

Some respondents reported that, other than antipsychotics for challenging behaviours, they are also withdrawing antidepressants, mood stabilisers and anti-epileptic drugs, among others. A systematic review found that antidepressants may make challenging behaviours worse in some adults with intellectual disabilities, possibly because of their adverse effects.^25^ Mood stabilisers reported by the respondents are likely to be lithium, as they separately reported about withdrawing anti-epileptics. Previous studies have reported a lower rate of lithium prescription,^17,26^ possibly because of its narrow therapeutic window, serious adverse effect profile and the difficulty in carrying out necessary blood tests, particularly in adults with severe and profound intellectual disabilities who show challenging behaviours.^7^ Where anti-epileptics have been withdrawn, it is not clear whether they were initially prescribed for challenging behaviours or epilepsy, as epilepsy is common in this population.^27^

### Behaviour change

A small proportion (11.4%) reported a deterioration in behaviour in over 50% of patients after withdrawal of antipsychotics. Interestingly, the same proportion (11.4%) reported no such deterioration. In the past, some studies showed either no change in behaviour or improvement in behaviour and quality of life on antipsychotic withdrawal in the majority of patients but others showed a worsening of behaviour in a high proportion of individuals.^7^ A number of studies showed that withdrawal of antipsychotics (particularly the older generation ones: chlorpromazine, haloperidol, thioridazine) may precipitate extrapyramidal symptoms, particularly dyskinesia, Parkinsonism and akathisia.^28^ These symptoms may be misinterpreted as challenging behaviours. Rebound akathisia might appear within the first few days, whereas rebound Parkinsonism usually emerges after a week and rebound dyskinesia might only become apparent within a month. However, most studies also show that these symptoms improve within a few weeks to months.^28^ This is an indicator for clinicians considering withdrawal of antipsychotics that, instead of reinstating antipsychotics straightaway because of the resurgence of challenging behaviours following antipsychotic withdrawal, they should wait (if necessary with the help of prescription when required (p.r.n.)) until the behaviour improves.^16^

### Factors associated with successful withdrawal

The factors described in Table 3 for a successful withdrawal are by and large the ones that have been reported in previous antipsychotic withdrawal studies.^17–19^ However, although some respondents mentioned antipsychotic monopharmacy as a helpful factor, a similar number suggested polypharmacy as helpful. This inconsistency is difficult to interpret and, given the small number, a definitive conclusion is challenging. It is also possible that the way this particular question was framed had scope for misinterpretation. These discrepancies highlight the complexity of the withdrawal process, which is influenced by so many internal and external factors.^28^

### Antipsychotics prescribed for mental illness

In total, 12.3% of respondents stated that over 50% of their patients who were receiving antipsychotics had a comorbid psychiatric illness. Therefore, they did not consider these patients for antipsychotic withdrawal. This proportion is very high, compared with the literature. For example, in a UK population-based study Sheehan and colleagues^11^ reported that 71% of adults with intellectual disabilities who were receiving antipsychotics (*n* = 9135) did not have a diagnosis of a severe mental illness. However, only 33.2% of respondents made the diagnosis of mental illness themselves and the rest depended on diagnosis made by others, including carer reports and GP records, and in many cases the diagnosis was historical and gathered from the case notes. The difficulty of making a diagnosis of a severe mental illness in adults with intellectual disabilities, particularly those who have severe and profound disability, is well known.^3,4,22^ It is possible that, although the diagnosis was made by others, the respondents agreed with it. However, this may leave room for error and misjudgement, leading to inappropriate use of medication as both false-positive and false-negative diagnosis of psychiatric illnesses is possible in people with intellectual disabilities, particularly when they show challenging behaviours.

It is not uncommon for adults with intellectual disabilities to have received a psychiatric diagnosis many years ago for which they have been receiving psychotropics for a long period without a review. Both national^9^ and international guidelines^10^ recommend a thorough mental state assessment of adults with intellectual disabilities who are receiving psychotropic medication for a diagnosed or suspected comorbid mental illness. This is even more important for those who received a psychiatric diagnosis historically (as the diagnostic criteria have changed over the years) and have been receiving psychotropics for a long time without a review.

### Outcome measures

The majority (67%) of respondents did not use any outcome measure to assess the effect of the antipsychotic withdrawal. Even for those who used some outcome measures, the type and quality/validity of these measures are not known. Both national^9,29^ and international^10^ guidelines recommend use of a validated outcome measure. At present there is no consistency across the UK in the type of outcome measures used, and this should be addressed by developing a national framework for psychiatrists, which should include recommendation on outcome measures.

### Barriers and support requirement for a successful antipsychotic withdrawal

Many respondents felt that the lack of multi-agency collaboration (22.4%) and multidisciplinary teamwork (20.1%) are major barriers in achieving withdrawal. It is also evident from the fact that a statistically significantly higher proportion of those respondents who reported having received support from their local services achieved a complete antipsychotic withdrawal compared with those who reported not having received such support. Multi-agency commitment and involvement are necessary to achieve NHS England's ‘transforming care’ goals.^30^ Unwin and colleagues highlighted the lack of input from the multi-agency team, particularly community nurses and clinical psychologists, in dealing with challenging behaviours in adults with intellectual disabilities.^31^ This issue is highlighted by the fact that many of our respondents felt that the lack of available non-pharmacological psychosocial intervention is often hindering the withdrawal process. Unwin and colleagues postulated that the lack of input from community nurses may have been caused by conflicting demand on their time to carry out many tasks that are not directly related to their professional role.^31^

### Carers’ role

Almost a quarter (22.4%) of respondents reported that concern felt by support staff and of family carers was a barrier to antipsychotic withdrawal. A number of authors have highlighted the influence of staff perception on the withdrawal process.^7,18^ In a recent survey of family carers’ views on the management of challenging behaviours in people with intellectual disabilities, carers expressed a wish to see a more person-centred multimodal approach to the management of challenging behaviours for their loved ones. They also highlighted the need for more involvement of family carers and the person with intellectual disability in the decision-making process, including decisions on the use of psychotropic medication. Some saw the benefit of psychotropic medication for challenging behaviours but others felt that currently in many areas there may be overreliance on such medication to manage challenging behaviours in people with intellectual disabilities.^32^ Many professionals and family carers feel that support staff would benefit from more knowledge and experience in dealing with medication for the management of challenging behaviours. This issue is addressed in a current project which is developing an online training programme for support staff and family carers (https://spectrom.wixsite.com/project).^33^ A family carers’ association, the Challenging Behaviour Foundation, has also developed online material for family carers (www.challengingbehaviour.org.uk).

### Recommendations

It is difficult to make robust recommendations on the basis of this small online survey. However, it highlights the need for a framework/structure for comprehensive medication review, with a view to withdrawing inappropriate medication, and training for psychiatrists and other stakeholders to support the implementation of the framework.

It is important to conduct a thorough assessment of challenging behaviours and of the person behind the behaviour, using an appropriate structure/framework to provide the right care.^34^ For example, consideration of medication must be set within the overall context of a person-centred care planning. This should involve all stakeholders, such as the care team members/professionals, support staff, family carers and, most importantly, the person with intellectual disability, from the outset in all stages of decision-making. Adherence to national and international guidance for rational prescribing must be monitored by the employers and other authorities in order to implement these guidelines in day-to-day practice. The need for non-pharmacological support and intervention should be continuously pursued.

Psychiatrists must be extra vigilant when assessing any previous or historical diagnosis of psychiatric illness in people with intellectual disabilities, given the difficulty of making a psychiatric diagnosis in this population. Furthermore, there must be a comprehensive support plan to address any deterioration in behaviour on withdrawal of medication. This plan has to be agreed at the outset and monitored on a regular basis by all stakeholders, including family carers, support staff and the person with intellectual disability.^32^

Psychiatric training also needs to consider key aspects of how to diagnose mental illnesses in people with intellectual disabilities in the presence of coexisting problems such as developmental disorders, attachment difficulties and trauma, particularly if there are no reliable informants with knowledge of development histories.

A holistic, multidisciplinary, multi-agency, person-centred approach should be adopted for a comprehensive medication review, to reduce overmedication in this population. Our study showed that two main barriers in implementing antipsychotic withdrawal were resistance from support staff and resistance from family carers. Therefore, training for support staff and family carers is essential (see https://spectrom.wixsite.com/project).

It would be worth repeating a similar survey in future to measure the impact of any innovations arising from this paper and any related future work.

### Strengths

This is the first attempt to collect data directly from UK psychiatrists to assess the effect of STOMP on their psychotropic prescribing practice. The survey also provides a reflection of real-life practice, gathering the experience of psychiatrists that can focus further work that aims to minimise use of antipsychotics for challenging behaviours.

### Weaknesses

The estimated return rate of 39% is low but not unexpected for this type of survey. It is possible that more psychiatrists who are engaged in antipsychotic withdrawal have responded to the online survey than those who are not. This may have introduced bias in the data. Some questions might be perceived as ambiguous and there may be some overlap between questions. Relying on psychiatrists’ retrospective reports and answers is likely to lead to approximations.

## Data Availability

The data that support the findings of this study are available from the corresponding author on reasonable request.

## References

[1] Smith S, Branford D, Collacott RA, Cooper SA, McGrother C. Prevalence and cluster typology of maladaptive behaviours in a geographically defined population of adults with learning disabilities. Br J Psychiatry 1996; 169: 219–27. 8871800 10.1192/bjp.169.2.219

[2] Deb S, Thomas M, Bright C. Mental disorder in adults with intellectual disability. 2: The rate of behaviour disorders among a community-based population aged between 16-64 years. J Intellect Disabil Res 2001; 45: 506–14. 11737537 10.1046/j.1365-2788.2001.00373.x

[3] Hemmings C, Deb S, Chaplin E, Hardy S, Mukherjee R. Research for people with intellectual disabilities and mental health problems: a view from the UK. J Ment Health Res Intellect Disabil 2013; 6: 127–58.

[4] Deb S, Bethea T, Havercamp S, Rifkin A, Underwood L. Disruptive, impulse-control, and conduct disorders In Diagnostic Manual – Intellectual Disability: A Textbook of Diagnosis of Mental Disorders in Persons with Intellectual Disability (2nd edn) (eds R Fletcher, J Barnhill, S-A Cooper): 521–60. NADD Press, 2016.

[5] Lundqvist LO. Prevalence and risk markers of behavior problems among adults with intellectual disabilities: a total population study in Örebro County, Sweden. Res Dev Disabil 2013; 34: 1346–56. 23417139 10.1016/j.ridd.2013.01.010

[6] Sjgafoos J, Elkins J, Kerr M, Attwood T. A survey of aggressive behaviour among a population of persons with intellectual disability in Queensland. J Intellect Disabil Res 1994; 38: 369–81. 7949789 10.1111/j.1365-2788.1994.tb00417.x

[7] Deb S. Psychopharmacology In Handbook of Evidence-Based Practices in Intellectual and Developmental Disabilities (ed NN Singh): 347–381. Springer International Publishing, 2016.

[8] Didden R, Lindsay W, Lang R, Sigafoos J, Deb S, Wiersma J, Aggression In Handbook of Evidence-Based Practices and Developmental Disabilities (ed NN Singh): 727–50. Springer International Publishing, 2016.

[9] National Institute for Health and Care Excellence. Challenging Behaviour and Learning Disabilities: Prevention and Interventions for People with Learning Disabilities Whose Behaviour Challenges (NICE Guideline NG11). NICE, 2015. 26180881

[10] Deb S, Kwok H, Bertelli M, Salvador-Carulla L, Bradley E, Torr J, International guide to prescribing psychotropic medication for the management of problem behaviours in adults with intellectual disabilities. World Psychiatry 2009; 8: 181–6. 19812757 10.1002/j.2051-5545.2009.tb00248.x PMC2758582

[11] Sheehan R, Hassiotis A, Walters K, Osborn D, Strydom A, Horsfall L. Mental illness, challenging behaviour, and psychotropic drug prescribing in people with intellectual disability: UK population-based cohort study. BMJ 2015; 351: h4326. 26330451 10.1136/bmj.h4326 PMC4556752

[12] Marston L, Nazareth I, Petersen I, Walters K, Osborn DPJ. Prescribing of antipsychotics in UK primary care: a cohort study. BMJ Open 2014; 4: e006135. 10.1136/bmjopen-2014-006135 PMC4281533 25524544

[13] de Kuijper G, Hoekstra P, Visser F, Scholte FA, Penning C, Evenhuis H. Use of antipsychotic drugs in individuals with intellectual disability (ID) in the Netherlands: prevalence and reasons for prescription. J Intellect Disabil Res 2010; 54: 659–67. 20426795 10.1111/j.1365-2788.2010.01275.x

[14] Glover G, Williams R, Branford D, Avery R, Chauhan U, Hoghton M, Prescribing of psychotropic Drugs to People with Learning Disabilities and/or Autism by General Practitioners in England (Technical Report). Public Health England, 2015.

[15] Ramerman L, Hoekstra PJ, de Kuijper G. Health-related quality of life in people with intellectual disability who use long-term antipsychotic drugs for challenging behaviour. Res Dev Disabil 2018; 75: 49–58. 29482036 10.1016/j.ridd.2018.02.011

[16] Branford D, Gerrard D, Saleem N, Shaw C, Webster A. Stopping over-medication of people with intellectual disability, autism or both (STOMP) in England part 1 – history and background of STOMP. Adv Mental Health Intellect Disabil 2019; 13: 31–40.

[17] Branford D. Factors associated with successful or unsuccessful withdrawal of antipsychotic drug therapy prescribed for people with learning disabilities. J Intellect Disabil Res 1996; 40: 322–9. 8884587

[18] Ahmed Z, Fraser W, Kerr MP, Kiernan C, Emerson E, Robertson J, Reducing antipsychotic medication in people with a learning disability. Br J Psychiatry 2000; 176: 42–6. 10789325 10.1192/bjp.176.1.42

[19] de Kuijper G, Evenhuis H, Mindera RB, Hoekstra PJ. Effects of controlled discontinuation of long-term used antipsychotics for behavioural symptoms in individuals with intellectual disability. J Intellect Disabil Res 2014; 58: 71–83. 23046144 10.1111/j.1365-2788.2012.01631.x

[20] de Kuijper GM, Hoekstra PJ. An open-label discontinuation trial of long-term, off-label antipsychotic medication in people with intellectual disability: determinants of success and failure. J Clin Pharmacology 2018; 58: 1418–26. 10.1002/jcph.1271 29920689

[21] Shankar R, Wilcock M, Deb S, Goodey R, Corson E, Pretorius C, A structured programme to withdraw antipsychotics among adults with intellectual disabilities: the Cornwall experience. J Appl Res Intellect Disabil 2019; 32: 1389–400. 31192534 10.1111/jar.12635

[22] Deb S, Matthews T, Holt G, Bouras N. Practice Guidelines for the Assessment and Diagnosis of Mental Health Problems in Adults with Intellectual Disability. Pavilion, 2001.

[23] Radouco-Thomas M, Bolduc M, Brisson A, Brassard P, Fortier L, Thivierge J. Pilot study on the use of psychotropic medication in persons with mental retardation. Prog Neuropsychopharmacol Biol Psychiatry 2004; 28: 879–83. 15363610 10.1016/j.pnpbp.2004.05.029

[24] Spreat S, Conroy JW, Fullerton A. Statewide longitudinal survey of psychotropic medication use for persons with mental retardation: 1994 to 2004. Am J Mental Retard 2004; 109: 322–31. 10.1352/0895-8017(2004)109<322:SLSOPM>2.0.CO;2 15176915

[25] Sohanpal SK, Deb S, Thomas C, Soni R, Lenôtre L, Unwin G. The effectiveness of antidepressant medication in the management of behaviour problems in adults with intellectual disabilities: a systematic review. J Intellect Disabil Res 2007; 51: 750–765. 17803494 10.1111/j.1365-2788.2006.00935.x

[26] Deb S, Unwin G, Deb T. Characteristics and the trajectory of psychotropic medication use in general and antipsychotics in particular among adults with an intellectual disability who exhibit aggressive behaviour. J Intellect Disabil Res 2015; 59: 11–25. 24450426 10.1111/jir.12119

[27] Berney T, Deb S. Epilepsy in Learning Disability In Oxford Textbook of Epilepsy and Epileptic Seizures (eds S Shorvon, R Guerrini, M Cook, S Lahtoo): 195–9. Oxford University Press, 2012.

[28] Deb S, Bertelli MO, Rossi M. Psychopharmacology In Textbook of Psychiatry for Intellectual Disability and Autism Spectrum Disorder (eds MO Bertelli, S Deb, K Munir, A Hassiotis, L Salvador-Carulla). Springer International Publishing, 2021 (in press).

[29] Faculty of Psychiatry of Intellectual Disability. Psychotropic Drug Prescribing for People with Intellectual Disability, Mental Health Problems and/or Behaviours that Challenge: Practice Guidelines (Faculty Report 2016 FR/ID/09). Royal College of Psychiatrists, 2016.

[30] Department of Health. Transforming Care: A National Response to Winterbourne View Hospital. Department of Health Review: Final Report. Department of Health, 2012.

[31] Unwin GL, Deb S, Deb T. Community-based specialist health service provision for the management of aggressive behaviour in adults with intellectual disabilities: an exploration of costs, medication and contacts with professionals. J Appl Res Intellect Disabil 2017; 30: 316–25. 26970410 10.1111/jar.12241

[32] Sheehan R, Kimona K, Giles A, Cooper V, Hassiotis A. Findings from an online survey of family carer experience of the management of challenging behaviour in people with intellectual disabilities, with a focus on the use of psychotropic medication. Br J Learn Disabil 2018; 46: 82–91.

[33] Deb S, Limbu B, Crawford M, Weaver T. Short-term PsychoEducation for Carers To Reduce Over Medication of people with intellectual disabilities (SPECTROM): study protocol. BMJ Open 2020; 10: e037912. 10.1136/bmjopen-2020-037912 PMC7245413 32273322

[34] Deb S, Bethea T, Havercamp S, Rifkin A, Underwood L. Disruptive, impulse-control, and conduct disorders In Diagnostic Manual – Intellectual disability: A textbook of Diagnosis of Mental Disorders in Persons with Intellectual Disability (2nd edn) (eds R Fletcher, J Barnhill, S-A Cooper): 521–560. NADD Press, 2016.

