# Supplementary material for: UK psychiatrists’ experience of withdrawal of antipsychotics prescribed for challenging behaviours in adults with intellectual disabilities and/or autism
Source: BJPsych Open. 2020 Sep 17;6(5):e112. doi: 10.1192/bjo.2020.97 (PMC7576649; doi:10.1192/bjo.2020.97)
Supplement: Supplementary file 1 [file S2056472420000976sup001.zip › S2056472420000976sup010.pdf]

Withdrawal of Antipsychotics Prescribed for Behaviours that Challenge in People with Intellectual disability and/or Autism

8

* Required

The following questions are going to ask you for some basic demographic information.

1. What is your current medical position? *

Mark only one oval.

- ◯ Consultant
- ◯ Specialty doctor
- ◯ Higher trainee
- ◯ Core trainee
- ◯ Other trainee
- ◯ Foundation doctor
- ◯ Other: ______________________

2. Where do you currently practice? *

Mark only one oval.

- South West
- South East
- London
- Eastern
- Wales
- West Midlands
- East Midlands
- North West
- Yorkshire and the Humber
- North East
- Scotland
- Northern Ireland
- Other: ____________________

3. How many years have you been practicing in psychiatry of intellectual disability? *

Mark only one oval.

- Less than 10 years
- 10-19 years
- 20-29 years
- >30 years

4. Do you have a database of people with intellectual disabilities and/or autism (PwID/ASD) prescribed antipsychotics in the absence of a severe mental illness? *

Mark only one oval.

- Yes
- No

The following set of questions are going to ask you about your experience of reducing/withdrawing antipsychotic medication prescribed for behaviours that challenge (BtC).

5. When did you start to withdraw antipsychotics prescribed for BtC in PwID/ASD? *

Mark only one oval.

- If you do not prescribe antipsychotics for PwID/ASD please select this option and exit the questionnaire. Thank you for your contribution.
- Not yet
- <1 year ago
- 1-3 years ago
- 3-5 years ago
- >5 years ago

6. Have you attempted withdrawal of other psychotropic medication in PwID/ASD? (Yes - please specify which psychotropics/No) *

____

7. In what proportion of PwID/ASD have you attempted to withdraw antipsychotics used to manage BtC? *

Mark only one oval.

- 0%
- 1-25%
- 26-50%
- >50%
- Data Not Available
- Other: ____________________

8. If you have a database to identify those in which withdrawal has been attempted, could you give an approximate of how many people this is?

____

9. In the last 12 months in PwID/ASD where attempts have been made to withdraw antipsychotics, what proportion of PwID/ASD have you completely withdrawn from antipsychotics prescribed for BtC? *

Mark only one oval.

- 0%
- 1-25%
- 26-50%
- >50%
- Data Not Available
- Other: ____________________

10. If you have a database to identify those in which withdrawal has been attempted, could you give an approximate of how many people this is?

____

11. In the last 12 months in PwID/ASD where attempts have been made to withdraw antipsychotics, in what proportion of people was a dose reduction of >50% achieved? *

Mark only one oval.

- 0%
- 1-25%
- 26-50%
- >50%
- Data Not Available
- Other: ______________________

12. If you have a database to identify those in which withdrawal has been attempted, could you give an approximate of how many people this is?

____

13. Of those in whom withdrawal was attempted but unsuccessful, approximately what proportion of people were reinstated on antipsychotics within: *

Mark only one oval per row.

	0%	1-25%	26-50%	>50%
3 months				
6 months				
12 months				

14. Of those in whom withdrawal was attempted, approximately what proportion of people were unsuccessful in withdrawal? *

Mark only one oval.

- ⬭ 0%
- ⬭ 1-25%
- ⬭ 26-50%
- ⬭ >50%
- ⬭ Data Not Available
- ⬭ Other: ____________

15. What proportion of people had a worsening of their status during attempted withdrawal of antipsychotics? (admitted to hospital, increase in medication, referral to crisis support or intensive support team) *

Mark only one oval.

- ⬭ 0%
- ⬭ 1-25%
- ⬭ 26-50%
- ⬭ >50%
- ⬭ Data Not Available
- ⬭ Other: ____________

16. Were there significant adverse consequences observed during withdrawal? *

Check all that apply.

- ☐ Worsening of BtC?
- ☐ Withdrawal symptoms
- ☐ Uncovering of underlying psychiatric symptoms

Other: ☐ ____________

17. Were any groups (e.g. those on antipsychotic monotherapy) more likely to successfully discontinue antipsychotic medication than others? *

Check all that apply.

- [ ] No
- [ ] Antipsychotic monotherapy
- [ ] Antipsychotic polypharmacy
- [ ] Psychotropic monotherapy
- [ ] Polypharmacy of psychotropics
- [ ] Polypharmacy including physical health drugs and psychotropics
- [ ] High dose antipsychotic
- [ ] Low dose antipsychotic
- [ ] Severe BtC
- [ ] Mild intellectual disability
- [ ] Moderate intellectual disability
- [ ] Severe intellectual disability
- [ ] Male
- [ ] Female
- [ ] Those living with family
- [ ] Those experiencing side effects of antipsychotic medication
- [ ] First time withdrawal attempt
- [ ] Previous failed withdrawal attempt

Other: [ ] ______________________

18. What percentage of your population on antipsychotics did you not review due to the presumption of rational prescribing of antipsychotics? i.e. a major mental illness for which antipsychotics are indicated *

Mark only one oval.

- 0%
- 1-25%
- 26-50%
- >50%
- Data Not Available
- Other: ______________________

19. How did you confirm the diagnosis of major mental illness and suitability of antipsychotics (choose all that apply) *

Check all that apply.

- [ ] I made the diagnosis
- [ ] The diagnosis was made by a colleague
- [ ] The diagnosis was historical and documented in the case notes
- [ ] A family member/carer/patient themselves provided the diagnosis
- [ ] The diagnosis was documented in the GP records

Other: [ ] ______________________

20. In those who you did not review the antipsychotic(s) how confident are you of their diagnosis of existing mental illness? *

Mark only one oval.

	1	2	3	4	5	
Not at all	○	○	○	○	○	Completely

The following set of questions are going to ask you about your prescribing habits since the introduction of the STOMP initiative in 2016.

21. Has STOMP made you less likely to start an antipsychotic in PwID/ASD for BtC? *

Mark only one oval.

- Yes
- No
- No difference

22. Since the introduction of the STOMP initiative, are you now more often prescribing other classes of psychotropic medication instead of antipsychotics for the pharmacological management of BtC? *

Mark only one oval.

- Yes
- No

23. If you answered yes to the previous question, what classes of medication are you now considering?

____

24. What factors make you more likely to initiate withdrawal from antipsychotics prescribed for BtC? Please state in the order of importance. *

____

____

____

____

____

The following set of questions are going to ask about structures in place to support the withdrawal of antipsychotics prescribed for BtC in PwID/ASD.

25. How successful do you think your area has been in adhering to the behaviour that challenges national guidance? *

Mark only one oval.

- Not at all
- A little
- A lot
- Completely

26. Has your local ID MDT received formal training on STOMP? (e.g. in the form of a presentation) *

Mark only one oval.

- Yes
- No

27. On a scale of 1-5 how confident are you in withdrawing antipsychotics prescribed for BtC in PwID/ASD? *

Mark only one oval.

	1	2	3	4	5	
Not at all						Completely confident

28. Why do you give this score?

29. Do you use outcome measures to monitor antipsychotic withdrawal? e.g. HoNOS-LD *

Mark only one oval.

- Yes
- No

30. Have you developed other resources or innovations to aid the antipsychotic withdrawal process? (e.g. access to STOMP leaflets or local experts by experience) *

31. Is there professional support available to aid withdrawal attempts? *

Check all that apply.

- [ ] No
- [ ] Multi-agency
- [ ] Shared care with general practice
- [ ] Multidisciplinary team
- [ ] Nursing
- [ ] Psychology
- [ ] Pharmacy
- [ ] Allied health professions

Other: [ ] ______________________

The following questions are going to ask you about the challenges you have faced and the successes you have experienced in withdrawing antipsychotics prescribed for BtC in PwID/ASD.

32. In your experience, what are the barriers and challenges to implementing the withdrawal of antipsychotics prescribed to PwID/ASD for BtC? *

Check all that apply.

- [ ] Competence in withdrawing antipsychotic medication
- [ ] Lack of guidance on the structured withdrawal of antipsychotics
- [ ] Accessing the support of the multidisciplinary team
- [ ] Resistance from staff involved in the care of the PwID/ASD to withdrawal
- [ ] Resistance from family of the PwID/ASD to withdrawal
- [ ] Lack of non-pharmacological options as alternatives to medication
- [ ] The lack of pharmacist input specifically to focus on psychotropic drugs
- [ ] The local GPs are reluctant to become involved in any STOMP initiative

33. Please list other challenges or barriers you have faced that are not listed above.

34. Which of the following extra resources do you believe would be most beneficial to improving the practice on antipsychotic prescribing for BtC in your area? Please select the top 3 most beneficial options. *

Check all that apply.

- [ ] An increase in multiagency working (GPs, social workers, pharmacists)
- [ ] An increase in multidisciplinary team working
- [ ] Access to a nurse prescriber
- [ ] Access to a pharmacist
- [ ] Access to a social worker
- [ ] Access to other allied health professions
- [ ] Financial help

35. What have been the biggest successes in the withdrawal of antipsychotics prescribed to PwID/ASD for BtC in your area?

36. Please give a 2-3 sentence summary of your experience in attempting the withdrawal of antipsychotics prescribed to PwID/ASD for BtC.

This content is neither created nor endorsed by Google.

Google Forms
